# The Helping by Clicking Types Questionnaire (HCTQ): The Development of a Measure to Assess Different Patterns of Helping by Clicking

**DOI:** 10.5964/ejop.10917

**Published:** 2024-08-30

**Authors:** Agata Błachnio, Aneta Przepiórka, Paweł Kot, Andrzej Cudo, Małgorzata Sobol

**Affiliations:** 1 Institute of Psychology, The John Paul II Catholic University of Lublin, Lublin, Poland; 2 Institute of Psychology, University of Warsaw, Warsaw, Poland; Università degli Studi di Bari Aldo Moro, Bari, Italy

**Keywords:** helping by clicking, helping on the Internet, behaviors on the Internet

## Abstract

Recently, there has been an increase in the number of aid campaigns launched via social media. The paper explores the phenomenon called “helping by clicking,” which consists in clicking “Like” to support a charitable campaign or cause. The main aim of the paper is to present a new measure: The Helping by Clicking Types Questionnaire (HCTQ), assessing the patterns of helping by clicking. In developing the questionnaire, we relied on the theory of reciprocal altruism. The study included two samples of *n* = 349 and *n* = 1,006 participants. The HTCQ consists of 19 items making up three subscales: People, Environment, and Animals. The present research included two independent studies. Study 1 was conducted to determine the psychometric properties of the questionnaire, while Study 2 was conducted to verify the previous results and to test the usefulness of the questionnaire in distinguishing individuals with different patterns of helping by clicking. The measure was designed to assess three aspects of helping: helping people, helping the environment, and helping animals via social media. The study showed that the HCTQ was suitable for measuring patterns of helping by clicking. All HCTQ factors had good Cronbach’s alpha reliability coefficients. The HCTQ concerns a new and largely unexplored area of helping that involves the use of modern technologies. It reveals people’s motivations for helping.

Recently, there has been an increase in the number of aid campaigns launched via social media (Demiroz & Akbas, 2022; Munroe et al., 2023). These campaigns consist, for example, in collecting “likes” for sick children, saving health, helping homeless animals, or supporting some other important cause. The information available often includes an account number where money can be donated. However, such help often comes down to clicking on a “Like” button. This may seem to be far from real help, but the phenomenon has become so popular that one can assume it is certainly not without significance for the people involved. This phenomenon is called helping by clicking, which means clicking “Like” to support a charitable campaign (see Wallace et al., 2017; Weiss & Cohen, 2019).

Helping behavior is understood within the framework of prosocial behavior and defined as providing support to others when this is not part of one’s job (Hafenbrack et al., 2022) or as doing something beneficial for others rather than for oneself (Twenge et al., 2007). It is explained by the theory of reciprocal altruism (Liu et al., 2023; Trivers, 1971), which accounts for helping behavior among unrelated individuals, and by the idea of a link between helping and emotions, amounting to a “doing good, feeling good” effect (e.g., Glomb et al., 2011). According to the theory of reciprocal altruism, helping others is beneficial from the evolutionary point of view. When helping others, one expects that they will return the favor, thus facilitating one’s survival.

The model of helping behavior that we rely on (see Aydinli et al., 2013) postulates the existence of two different mechanisms involved: *passive helping* is the outcome of a latent process automatically activated by affective components, whereas *active helping* is based to a greater extent on conscious and cognitive determinants and involves conscious and explicit effort. Based on this model, we distinguished four types of helping behavior. Active helping, engaging the helper to a greater extent and requiring greater effort from the helper in the form of time and expenditure (e.g., money), is subdivided into two types: (1) active charitable helping (aimed at supporting people who need help, for example by donating money) and (2) active irrational helping (helping people merely because they ask for help when pursuing their private goals or whims, such as buying a new brand of phone or traveling). Passive helping is understood as helping without making much effort, for example by taking and sharing photos and videos as a form of support or help; it comprises the following types of helping behavior: (3) passive charitable helping (clicking on links, sharing contents with a request for help from a well-known charity) and (4) passive irrational helping (clicking on aid campaign links or sharing contents in situations when private motives are irrelevant, only because someone is asking for help).

A phenomenon discussed in the literature similar to the one we have been analyzing is cyber-volunteering (Ahmed & Radwan, 2022). However, it involves remote performance of activities to the benefit of others via the Internet and social media through formal volunteering (Raja-Yusof et al., 2016). Surveys developed to date, such as the International Volunteer Impacts Survey (Lough et al., 2009), and studies on the importance of using electronic volunteering (Ahmed & Radwan, 2022) also refer for the most part to this phenomenon. The main focus of our interest has been the provision of help by common users of social media platforms, not engaged in any formal volunteering. The investigation of this phenomenon required the development of a relevant, valid, and reliable measure. The main aim of this paper is to present a new measure assessing patterns of helping by clicking (HCTQ). To our knowledge, our questionnaire is the first one to assess this phenomenon. Our goal was also to check whether it was possible to identify groups of individuals with different helping patterns using the new tool. In the existing studies, any attempts to structure the population of individuals offering help through social media have involved classification based on specific issues bringing together online volunteer groups in social media platforms (Demiroz & Akbas, 2022) rather than exploratory research on volunteers.

The present research included two independent studies, with two separate samples: Study 1 and Study 2. The aim of Study 1 was to determine the psychometric properties of the questionnaire being developed, particularly to test its factor structure. Study 2 was conducted to verify the previous results and to test the usefulness of the questionnaire in distinguishing individuals with different patterns of helping by clicking.

## Study 1

### Method

#### Participants and Procedure

Study 1 was conducted online, and the criterion for participation was social media activity. It included 349 participants (255 females; age range: 16–60 years, *M* = 28.07, *SD* = 8.51), who completed an online survey. The characteristics of the sample from Study 1 are presented in Table 1. Participation in Study 1 was voluntary, and participants were assured that their responses were anonymous. All the procedures applied were in accordance with the ethical standards of the responsible committee on human experimentation (institutional and national) and with the Helsinki Declaration. The dataset from Study 1 is available from Błachnio et al. (2024S-a).

**Table 1:** Sample 1 Characteristics – Study 1 (N = 349)

Variable / Category	*N*	Percentage	*M*	*SD*
Gender				
Female	255	73.07		
Male	94	26.93		
Residence				
Village	77	22.06		
Small town	53	15.19		
Medium-sized town	39	11.17		
Big city	180	51.58		
Number of hours of Internet usage on weekdays			7.04	5.52
Number of hours of Internet usage on weekends			8.11	6.03

#### Measures

Before the development of the Helping by Clicking Types Questionnaire (HCTQ), two researchers searched the Internet for charities where people could help by sharing and passing on information via the Internet and via social media. In the next step, they made a list of areas that could be supported via the Internet and social networking sites. Their work resulted in a list of 19 types of aid campaigns that can appear on the Internet and in social media (see Supplementary Materials; Table S1). Participants rated the HCTQ items on the following 7-point scale: 1 = *never*, 2 = *very rarely*, 3 = *rarely*, 4 = *sometimes*, 5 = *often*, 6 = *very often*, 7 = *always*.

#### Statistical Analysis

In Study 1, we computed correlation coefficients and descriptive statistics and performed an exploratory factor analysis (EFA) for Sample 1 (*N* = 349). The Pearson correlation coefficient was used to calculate the interrelations among the items. Additionally, we computed descriptive statistics: means (*M*), standard deviations (*SD*), skewness, and kurtosis. In order to determine the structure of the questionnaire, we performed an EFA using the principal component extraction method with Promax rotation. We also implemented the scree plot (Cattell, 1966) and the Kaiser criterion (Kaiser, 1960) to determine the number of factors. Multicollinearity and sampling adequacy were verified using the Kaiser–Meyer–Olkin criterion and Bartlett’s test of sphericity (Hair et al., 2010). The factor loadings were regarded as acceptable if they were above the threshold of .50 (Ferguson & Cox, 1993). We calculated the Cronbach alpha coefficient, McDonald's omega coefficient, composite reliability (CR), and average variance extracted (AVE) for each factor to provide an indication of internal consistency reliability.

We used SPSS 28 (IBM Corp., 2021) software with OMEGA macro (Hayes & Coutts, 2020) to compute descriptive statistics and internal consistency reliability indexes and to perform exploratory factor analysis, variance analysis, and correlation analysis. The scripts of statistical analyses performed in SPSS are presented in Supplementary Materials (see Table S2).

### Results

The analysis of correlation coefficients revealed that all correlations between items were statistically significant (see Table 2). Descriptive statistics—means (*M*), standard deviations (*SD*), skewness, and kurtosis—are presented in Table 3.

**Table 2:** Pearson Correlations Between Items (N = 349)

Item	1	2	3	4	5	6	7	8	9	10	11	12	13	14	15	16	17	18	19
1. Item 1	–																		
2. Item 2	.70	–																	
3. Item 3	.72	.59	–																
4. Item 4	.55	.75	.50	–															
5. Item 5	.63	.69	.62	.67	–														
6. Item 6	.61	.69	.54	.67	.68	–													
7. Item 7	.85	.60	.71	.55	.60	.62	–												
8. Item 8	.69	.87	.60	.76	.77	.74	.65	–											
9. Item 9	.89	.64	.70	.58	.62	.65	.89	.70	–										
10. Item 10	.67	.53	.89	.50	.62	.52	.72	.59	.70	–									
11. Item 11	.60	.52	.70	.60	.63	.51	.61	.56	.62	.74	–								
12. Item 12	.59	.74	.57	.72	.78	.66	.59	.81	.60	.57	.65	–							
13. Item 13	.68	.55	.83	.54	.66	.53	.70	.60	.68	.87	.81	.66	–						
14. Item 14	.69	.57	.85	.56	.63	.52	.71	.61	.70	.87	.80	.65	.92	–					
15. Item 15	.65	.51	.85	.55	.60	.50	.69	.56	.68	.89	.76	.58	.85	.88	–				
16. Item 16	.87	.61	.70	.56	.63	.59	.87	.66	.90	.70	.64	.61	.71	.74	.72	–			
17. Item 17	.82	.57	.64	.53	.55	.56	.82	.62	.87	.64	.59	.58	.66	.69	.66	.87	–		
18. Item 18	.64	.77	.61	.70	.76	.72	.61	.85	.65	.59	.55	.81	.63	.64	.59	.64	.64	–	
19. Item 19	.58	.68	.50	.64	.69	.68	.58	.76	.59	.48	.52	.72	.55	.53	.49	.57	.56	.78	–

*Note.* All correlations are statistically significant (*p* < .001).

**Table 3:** Descriptive Statistics, Exploratory Factor Analysis Results, and Internal Consistency Analysis Results (N = 349)

	Descriptive statistics				Factor loadings		
Item	*M*	*SD*	Skewness	Kurtosis	Factor 1	Factor 2	Factor 3
Item 1	3.38	1.91	0.220	−1.150			.851
Item 2	3.47	1.94	0.159	−1.226	.859		
Item 3	3.45	1.88	0.082	−1.210		.811	
Item 4	3.00	1.74	0.415	−0.857	.859		
Item 5	2.95	1.77	0.508	−0.834	.751		
Item 6	3.41	1.95	0.200	−1.180	.766		
Item 7	3.66	2.08	0.108	−1.306			.840
Item 8	3.48	1.87	0.133	−1.178	.896		
Item 9	3.40	2.02	0.265	−1.237			.902
Item 10	3.38	1.90	0.193	−1.179		.913	
Item 11	2.70	1.71	0.611	−0.764		.830	
Item 12	3.11	1.82	0.376	−0.938	.865		
Item 13	3.17	1.86	0.332	−1.031		.903	
Item 14	3.13	1.84	0.349	−1.073		.891	
Item 15	3.18	1.85	0.350	−1.004		.927	
Item 16	3.36	2.03	0.311	−1.195			.849
Item 17	3.27	2.03	0.356	−1.161			.900
Item 18	3.44	1.87	0.220	−1.001	.852		
Item 19	3.38	1.96	0.305	−1.096	.854		
Eigenvalue					12.99	1.78	1.10
Variance explained					68.35	9.36	5.76
Cronbach’s α					.96	.97	.97
McDonald's ω					.96	.97	.97
CR					.95	.95	.93
AVE					.70	.77	.75

The Kaiser–Meyer–Olkin measure of sampling adequacy (KMO = .962) and Bartlett’s test of sphericity, χ^2^(171) = 8789.88, *p*  < .001, confirmed the appropriateness of conducting the EFA (Hair et al., 2010). Considered against the scree plot criterion (Cattell, 1966) and the Kaiser criterion of eigenvalue above  1 (Kaiser, 1960), the EFA results pointed to a three-factor solution, which explained 83.48% of the total variance. All items had factor loadings above the acceptability threshold of .50 (see Table 3). In order to check whether the extracted factors represented one higher order factor, we repeated the EFA with factor scores as variables. Also in this case, the Kaiser–Meyer–Olkin measure of sampling adequacy (KMO = .735) and Bartlett’s test of sphericity, χ^2^(3) = 496.14, *p* < .001, confirmed the appropriateness of conducting the EFA (Hair et al., 2010). Considered against the scree plot criterion (Cattell, 1966) and the Kaiser criterion (eigenvalue = 2.36; Kaiser, 1960), the EFA yielded a single factor explaining 78.70% of the total variance in the higher order construct (Supplementary Materials; Table S3). It can therefore be assumed that the three extracted factors make up one higher order factor.

The values of Cronbach’s α and CR for each of the factors were above the desired threshold of .70. AVE was above the recommended threshold of .50 for each factor as well (see Table 3).

Additionally, the content analysis of the items indicated that Factor 1 concerned helping by clicking in the human context, Factor 2 consisted of items whose content related to helping by clicking in matters of the environmental, and the items making up Factor 3 referred to helping by clicking in the context of animals. Accordingly, Factor 1 was named Helping People by Clicking (abbreviated to People), Factor 2 was named Helping the Environment by Clicking (abbreviated to Environment), and Factor 3 was named Helping Animals by Clicking (abbreviated to Animals).

## Study 2

### Method

#### Participants and Procedure

The online survey in Study 2 was completed by 1,054 participants. However, 32 participants were excluded from the analyzed sample due to data gaps in surveys. Further 16 participants were excluded because they reported not using the Internet. Consequently, the final sample consisted of 1,006 participants (676 females; age range: 14–45 years, *M* = 19.54 years, *SD* = 2.93). The characteristics of the sample from Study 2 are presented in Table 4. Participation in Study 2 was also voluntary, and participants were assured that their responses were anonymous. All the procedures applied were in accordance with the ethical standards of the responsible committee on human experimentation (institutional and national) and with the Helsinki Declaration. The dataset from Study 2 is available from from Błachnio et al. (2024S-b).

**Table 4:** Sample 2 Characteristics – Study 2 (N = 1,006)

Variable / Category	*N*	Percentage	*M*	*SD*
Gender				
Female	676	67.20		
Male	330	32.80		
Residence				
Village	354	35.19		
Small town	214	21.27		
Big city	438	43.54		
Number of hours of Internet usage on weekdays			7.19	5.68
Number of hours of Internet usage on weekends			8.34	5.73

#### Measures

Study 2 used the Helping by Clicking Types Questionnaire (HCTQ) developed in Study 1. The HCTQ consisted of 19 items to which participants responded using a 7-point scale: 1 = *never*, 2 = *very rarely*, 3 = *rarely*, 4 = *sometimes*, 5 = *often*, 6 = *very often*, 7 = *always*. The reliability of the subscales, assessed using Cronbach's α, was .96 for the Animals subscale, .96 for the People subscale, and .96 for the Environment subscale.

#### Statistical Analysis

In Study 2 (*N* = 1,006), we performed a confirmatory factor analysis (CFA), item responses theory analysis (IRT), and latent profile analysis (LPA). CFA was performed to test if the structure of the HCTQ in Sample 2 was identical to the structure found in Sample 1. We used the maximum likelihood method with Satorra–Bentler correction (Satorra & Bentler, 1994). The following statistics were applied as measures of model fit in CFA: χ^2^, χ^2^/*df*, root mean square error of approximation (RMSEA), standardized root mean square residual (SRMR), comparative fit index (CFI), and Tucker-Lewis index (TLI; Kline, 2011). SRMR values lower than .08 indicate acceptable model fit. RMSEA values lower than .05 indicate good fit, and RMSEA values between .05 and .08 indicate moderate fit (see MacCallum et al., 1996). Additionally, χ^2^/*df* values below 3 indicate acceptable model fit (Kline, 2011). However, Marsh and Hocevar (1985) pointed out that χ^2^/*df* values below 5 indicated a reasonable fit. CFI and TLI values higher than .90 allow for concluding that a model acceptably fits a data set (Hu & Bentler, 1999; Kline, 2011).

Considering that CFA does not constitute an exhaustive analysis at the item level, we performed an item response theory (IRT) analysis, reflecting the connection between the underlying psychological construct being measured and the measurement process (Hambleton & Swaminathan, 2013). Consequently, IRT was used to explain the connection between the constructs assessed by the HCTQ and their manifestations in particular items. Considering the ordinal responses scale used in the HCTQ, we carried out a graded response model (GRM) analysis (Samejima, 1997) separately for each of the HCTQ subscales. This polytomous IRT model yields two types of item parameter estimates: the item discrimination parameter (α) and the item difficulty parameter (β) (see Goodman, 1974; Hambleton & Swaminathan, 2013; Samejima, 1997). Additionally, to analyze the items in the HCTQ subscales more accurately, we used the item response category characteristic curve (CCC). The CCC demonstrated the probability of individuals choosing a certain response on the scale at various levels of the latent variable representing different patterns of helping by clicking (see Hambleton & Swaminathan, 2013).

In order to examine if it was possible to distinguish groups with different types of helping by clicking using the HCTQ, we performed a latent profile analysis (LPA). The identification of individuals’ latent profiles was based on the three HCTQ subscales (People, Environment, and Animals). LPA is a statistical method using continuous observed variables to identify individuals’ unmeasured class membership. It should be noted that class membership is unknown but can be deduced from a series of continuous observed variables. This method focuses on relations among individuals to sort them into coherent groups that are different from one another. Because the analysis aimed to isolate independent profiles reflecting different types of helping by clicking, the model did not estimate covariances, and the variances were assumed to be the same across the profiles (see Rosenberg et al., 2021). The Akaike information criterion (AIC), consistent Aikake information criterion (CAIC), approximate weight of evidence (AWE), Bayesian information criterion (BIC), sample size-adjusted Bayesian information criterion (SABIC), classification likelihood criterion (CLC), the Kullback information criterion (KIC), and entropy (see Akogul & Erisoglu, 2017) were used for model selection decisions. To better describe the profiles, we performed a two-way repeated measures ANOVA with Dunnett's T3 and Bonferroni post hoc tests. In the ANOVA, class membership was a between-subjects factor and HCTQ subscale was a within-subjects factor. Additionally, we used partial eta squared ($\eta_{p}^{2}$) to assess effect size. Scripts of statistical analyses performed in SPSS are presented in Supplementary Materials (see Table S5).

Stata 15 software (StataCorp., 2017) was used to calculate confirmatory factor analysis, variance analysis, and response item theory (IRT) analysis. Jamovi 2.3 (Jamovi Project, 2022) with tidyLPA package (Rosenberg et al., 2021) was used to perform latent profile analysis (LPA).

### Results

**Figure 1:**
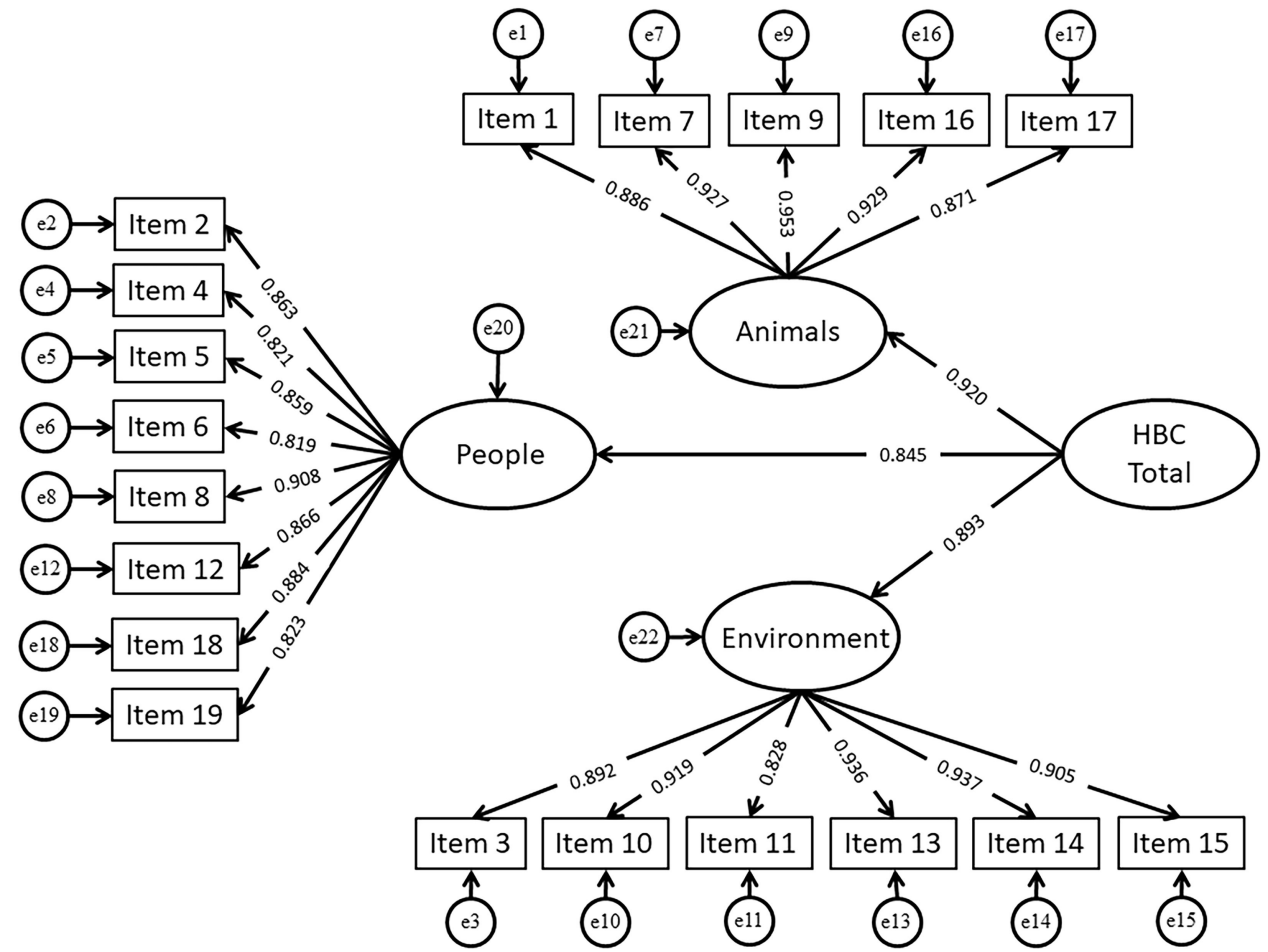
Confirmatory Factor Analysis Results *Note.* HBC = Helping by clicking. All paths are statistically significant (*p* < .001).

CFA results for Sample 2 (*N* = 1,006) showed that the three-factor model with a higher order factor was reasonably fitted to the data: χ^2^(*df* = 149) = 855.90, χ^2^/*df =* 5.74, *p* < .001, RMSEA = .069, SRMR = .033, CFI = .949, TLI = .941. All standardized factor loadings were statistically significant (*p* < .001), and their values ranged from .819 to .953. Detailed findings are presented in Figure 1.

For IRT analysis, the item discrimination parameter (α) was above zero for all items, which may indicate that all items effectively discriminate different levels of the latent constructs associated with the types of helping by clicking (people, environment, animals; see Table 5). Additionally, the item location threshold values (β) reflected the levels of latent variables (θ) at which the probability of choosing an answer below and above the threshold was equal (see Table 6). This may indicate that the response pattern obtained using the response scale for each item corresponds to the distribution of the latent variables that the items assess (Yang & Kao, 2014).

**Table 5:** IRT Analysis Results (N = 1,006)

Item	α	β_1_	β_2_	β_3_	β_4_	β_5_	β_6_
People subscale							
Item 2	3.67	−1.41	−1.08	−0.72	−0.23	0.29	0.85
Item 4	3.26	−1.20	−0.77	−0.49	0.17	0.72	1.30
Item 5	3.64	−1.24	−0.84	−0.48	0.14	0.74	1.36
Item 6	3.00	−1.46	−1.03	−0.71	−0.22	0.39	1.01
Item 8	4.76	−1.45	−1.05	−0.77	−0.29	0.26	0.87
Item 12	3.73	−1.39	−0.94	−0.63	−0.01	0.62	1.25
Item 18	4.14	−1.46	−1.02	−0.71	−0.14	0.49	1.10
Item 19	3.13	−1.38	−1.03	−0.74	−0.19	0.38	0.96
Environment subscale							
Item 3	4.04	−1.39	−1.04	−0.67	−0.16	0.39	0.95
Item 10	5.03	−1.38	−1.05	−0.69	−0.21	0.33	0.84
Item 11	3.33	−1.17	−0.79	−0.46	0.16	0.71	1.38
Item 13	6.84	−1.23	−0.92	−0.59	−0.11	0.44	0.94
Item 14	7.05	−1.25	−0.93	−0.57	−0.07	0.48	0.98
Item 15	4.89	−1.33	−0.96	−0.59	−0.02	0.50	1.06
Animals subscale							
Item 1	4.38	−1.30	−1.00	−0.71	−0.28	0.23	0.66
Item 7	5.73	−1.34	−1.08	−0.85	−0.40	0.03	0.49
Item 9	8.35	−1.25	−0.98	−0.73	−0.26	0.16	0.62
Item 16	6.06	−1.29	−0.97	−0.73	−0.31	0.13	0.66
Item 17	3.82	−1.23	−0.95	−0.69	−0.18	0.34	0.87

**Table 6:** Results of Model Selection in Latent Profile Analysis (N = 1,006)

									Number of Individuals in Each Class								
Solution	AIC	AWE	BIC	CAIC	CLC	KIC	SABIC	Entropy	Class 1	Class 2	Class 3	Class 4	Class 5	Class 6	Class 7	Class 8	Class 9
1-class	11666	11753	11696	11702	11656	11675	11677	1	1006								
2-class	10062	10209	10111	10121	10044	10075	10080	0.955	832	174							
3-class	9522	9728	9591	9605	9496	9539	9546	0.841	531	343	132						
4-class	9389	9655	9478	9496	9355	9410	9421	0.770	318	199	366	123					
5-class	9280	9605	9388	9410	9238	9305	9318	0.805	233	70	236	104	363				
6-class	9117	9500	9244	9270	9066	9146	9162	0.851	250	72	231	102	309	42			
7-class	9080	9523	9227	9257	9021	9113	9132	0.871	191	77	166	198	108	223	43		
8-class	8961	9463	9128	9162	8895	8998	9020	0.913	191	42	66	196	87	229	38	157	
9-class	8957	9519	9144	9182	8883	8998	9023	0.856	181	61	27	10	192	93	228	54	160

**Figure 2:**
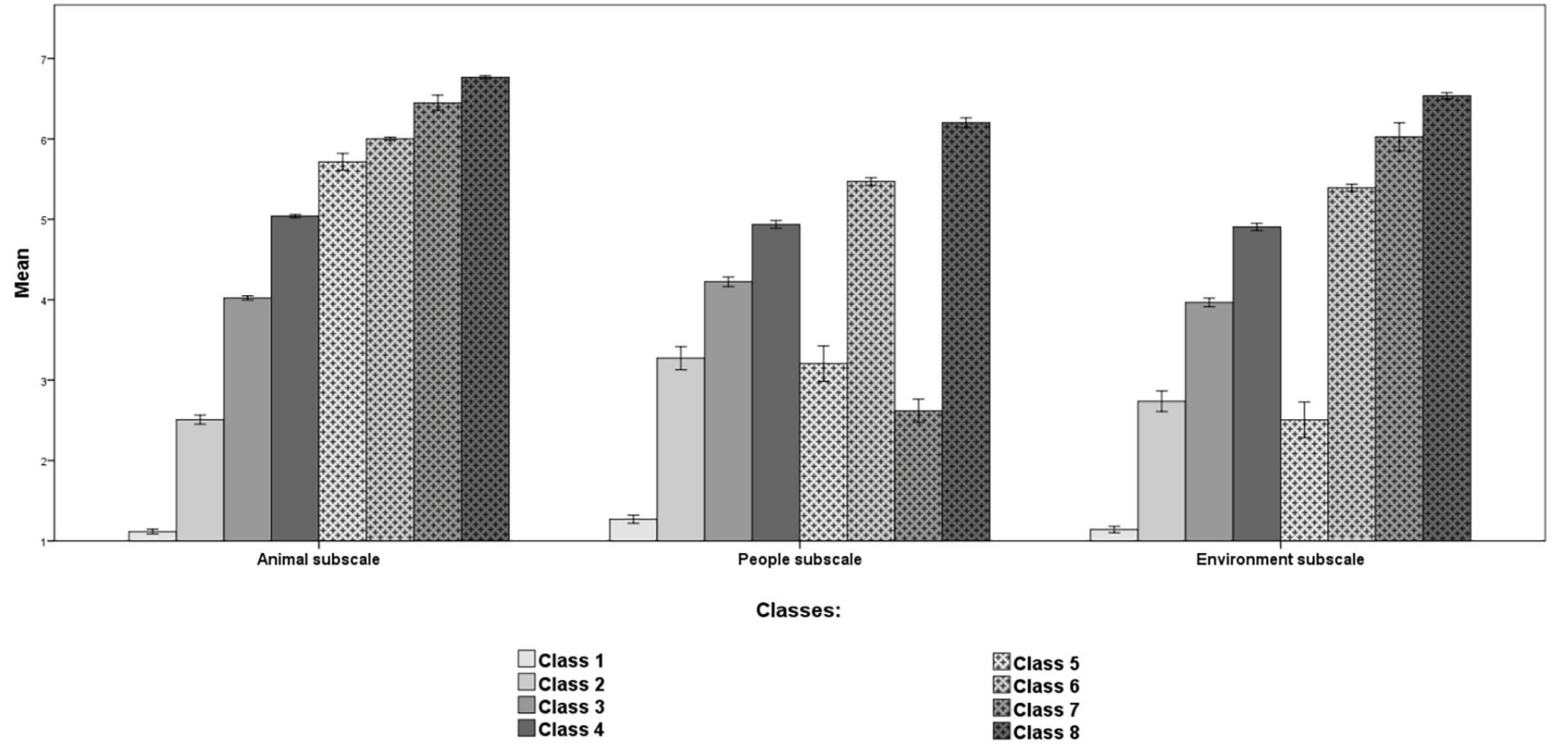
Item Response Category Characteristic Curves (CCC) for the Eight Items of the People Subscale

**Figure 3:**
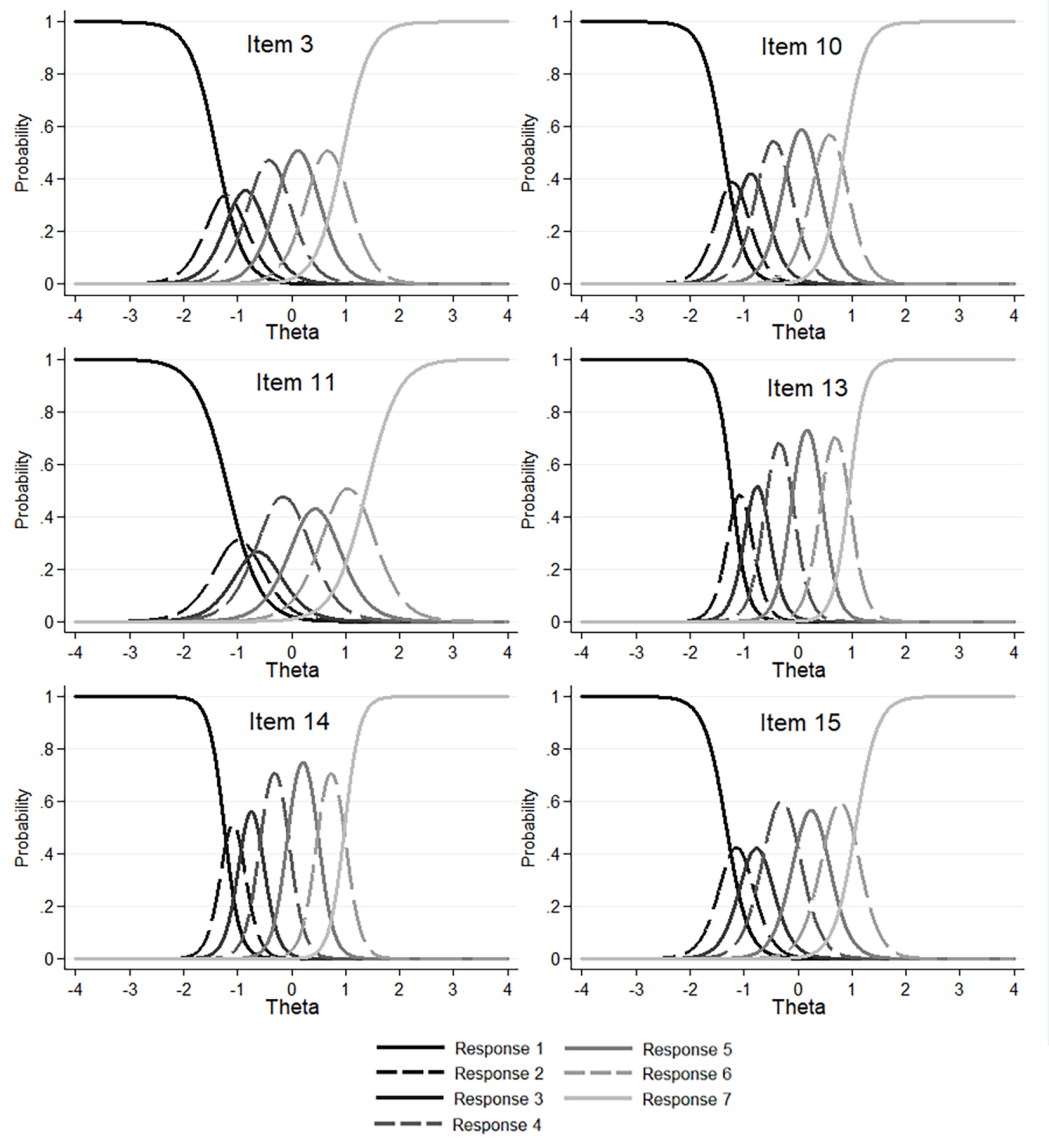
Item Response Category Characteristic Curves (CCC) for the Six Items of the Environment Subscale

**Figure 4:**
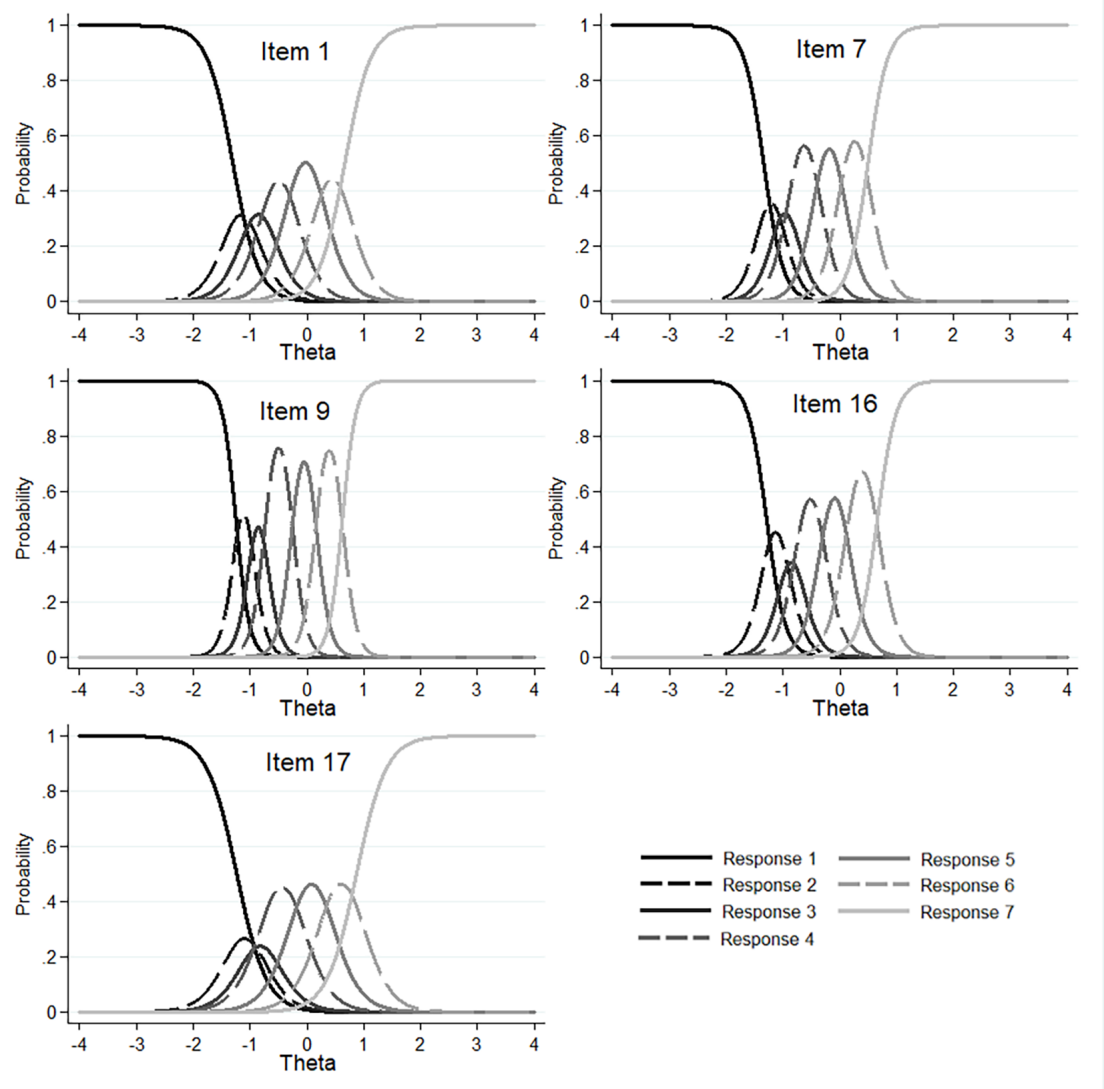
Item Response Category Characteristic Curves (CCC) for the Five Items of the Animals Subscale

For the item response category characteristic curve (CCC), the results in the form of graphs are presented separately for the People subscale (see Figure 2), the Environment subscale (Figure 3), and the Animals subscale (see Figure 4). Each curve in each plot reflects the probability of a given response to a given item as a function of the latent trait (θ). The results showed that the curves relating to the second (*very rarely*) and third (*rarely*) responses often overlapped with those relating to the first (*never*) and fourth (*sometimes*) responses (see Figures 2, 3, and 4). This indicates that the second (*very rarely*) and third (*rarely*) responses may be less distinguishable for participants than the remaining ones.

Regarding the identification of groups of individuals with different helping patterns, as lower values of AIC, AWE, BIC, CAIC, CLC, KIC, and SABIC and higher values of entropy indicate a better model, the analyses revealed that the 8-Class model was the best fitted one. However, it should be noted that Class 2 (*N* = 42, 4.2%) and Class 7 (*N* = 38, 3.8%) accounted for less than 5% of the total sample which means these classes should be interpreted with caution.

The results of two-way repeated measures ANOVA revealed a main effect of class membership, *F*(7, 998) = 1548.14, *p* < .001, $\eta_{p}^{2}$ = .92. Dunnett's T3 post hoc test showed that almost all pairwise comparisons between classes were significant (*p* < .001), except for two pairs: Class 7 vs. Class 4 (*p* = .519). The classes scored as follows on helping by clicking: Class 1 *M* = 6.32, *SE* = 0.03; Class 2 *M* = 2.44, *SE* = 0.07; Class 3 *M* = 3.32, *SE* = 0.06; Class 4 *M* = 4.90, *SE* = 0.03; Class 5 *M* = 1.14, *SE* = 0.05; Class 6 *M* = 5.52, *SE* = 0.03; Class 7 *M* = 4.66, *SE* = 0.07; Class 8 *M* = 4.10, *SE* = 0.04. There was also a main effect of HCTQ subscales, *F*(2, 997) = 79.70, *p* < .001, $\eta_{p}^{2}$ = .14. The Bonferroni post hoc test showed that there was a statistically significant difference between the Animals subscale and the People subscale (*p* < .001), and between animal subscale and environment subscale (*p* < .001). The participants were more likely to help by clicking when the post was about animals (*M* = 4.28, *SE* = 0.01) than when it was about people (*M* = 3.91, *SE* = 0.03). They reported helping by clicking less often when the post was about the environment (*M* = 3.96, *SE* = 0.04) than when it was about the animals (*M* = 4.28, *SE* = 0.01). There was no significant difference between the People and Environment subscales (*p* = .840).

There was a first-order class membership × HCTQ subscales interaction, *F*(14, 1996) = 51.32, *p* < .001, $\eta_{p}^{2}$ = .27. The results of pairwise comparisons between HCTQ subscales within each class are presented in Table 7. Additionally, the results revealed different patterns of differences between HCTQ subscales according to class membership. Detailed findings from class membership pairwise comparisons for each HCTQ subscale are presented in Supplementary Materials (Table S5). Figure 2 shows the results for each condition; however, for the purpose of clarity, information on statistical significance is not included (see Table 7).

**Table 7:** Results of Pairwise Comparisons Between HCTQ Subscales on the Class Level (N = 1,006)

	Pairwise comparisons						
Class	HCTQ subscales	*M*	*SE*	HCTQ subscales	*M*	*SE*	*p*
Class 1	Animals	6.83	0.02	People	5.98	0.06	.000
	Animals	6.83	0.02	Environment	6.15	0.07	.000
	People	5.98	0.06	Environment	6.15	0.07	.133
Class 2	Animals	1.82	0.04	People	2.80	0.13	.000
	Animals	1.82	0.04	Environment	2.71	0.14	.000
	People	2.80	0.13	Environment	2.71	0.14	1.000
Class 3	Animals	2.95	0.03	People	3.81	0.10	.000
	Animals	2.95	0.03	Environment	3.19	0.11	.123
	People	3.81	0.10	Environment	3.19	0.11	.000
Class 4	Animals	4.99	0.02	People	4.87	0.06	.184
	Animals	4.99	0.02	Environment	4.85	0.07	.152
	People	4.87	0.06	Environment	4.85	0.07	1.000
Class 5	Animals	1.05	0.03	People	1.21	0.09	.273
	Animals	1.05	0.03	Environment	1.17	0.10	.729
	People	1.21	0.09	Environment	1.17	0.10	1.000
Class 6	Animals	5.90	0.02	People	5.40	0.06	.000
	Animals	5.90	0.02	Environment	5.27	0.06	.000
	People	5.40	0.06	Environment	5.27	0.06	.213
Class 7	Animals	6.65	0.04	People	3.04	0.14	.000
	Animals	6.65	0.04	Environment	4.29	0.15	.000
	People	3.04	0.14	Environment	4.29	0.15	.000
Class 8	Animals	4.08	0.02	People	4.17	0.07	.528
	Animals	4.08	0.02	Environment	4.04	0.07	1.000
	People	4.17	0.07	Environment	4.04	0.07	.448

**Figure 5:**
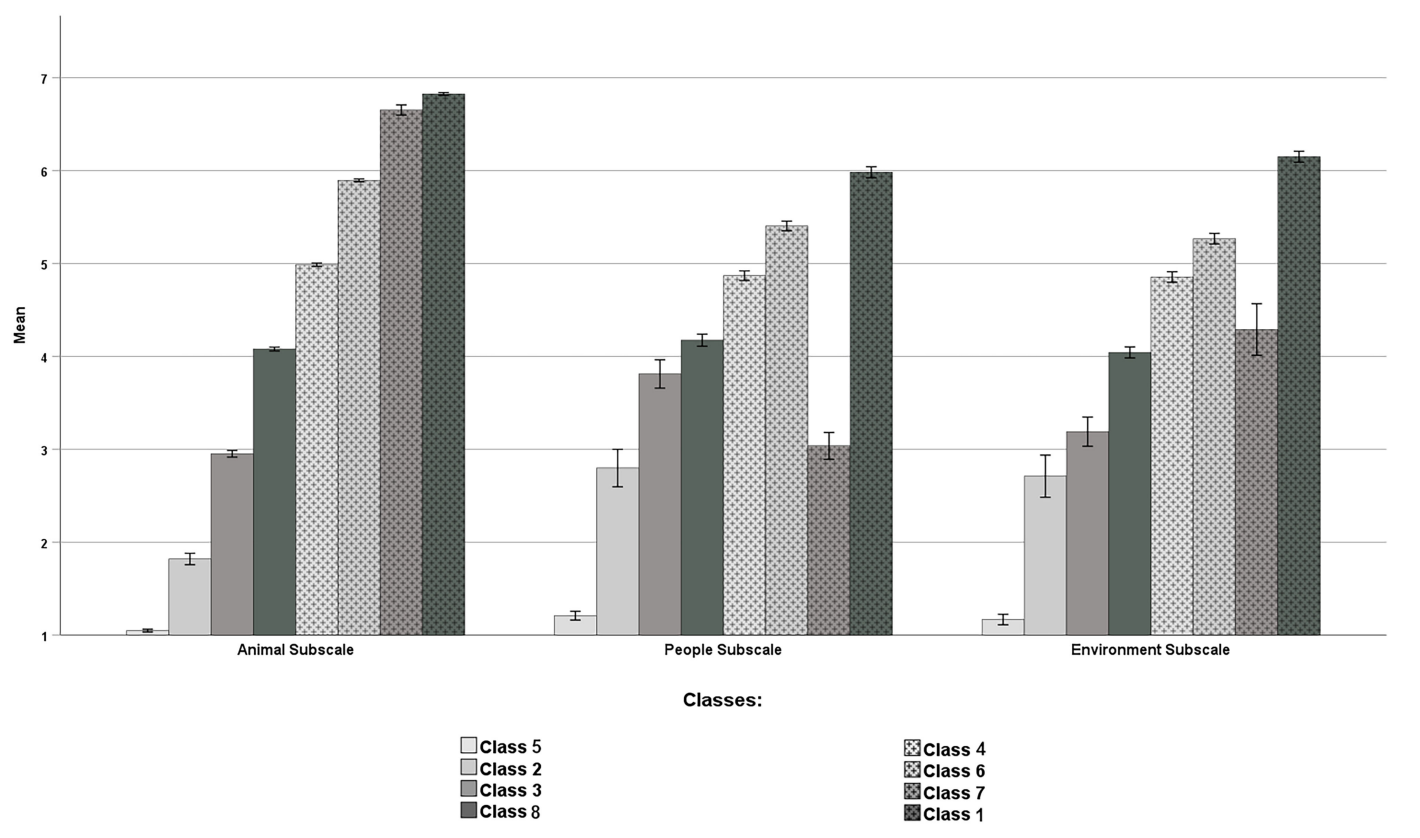
Helping by Clicking Scores on HCTQ Subscales and Class Membership Levels

The classes distinguished based on LPA are differentiated by the type of helping by clicking reported by their members. More specifically, Class 5 and Class 2 can be described as low helpers in every aspect of helping by clicking (see Figure 5). However, individuals in Class 2 were more willing to help by clicking when posts were about people than in the case of posts related to animals and the environment (see Table 7). Class 3, Class 4, and Class 8 can be designated as medium helpers. Individuals in Class 3 were more likely to help by clicking when posts were about people rather than animals or the environment. However, individuals in Class 4 and Class 8 did not exhibit a difference between the areas of helping by clicking (see Table 7). Finally, Class 1, Class 6, and Class 7 can be described as high helpers. It should be noted that there are large differences among these groups. Individuals in Class 1 helped by clicking primarily when posts were about animals, but they very rarely helped in response to posts about people or the environment. Similarly, subjects in Class 6 also preferred helping animals, but contributed to a similar average degree when posts were about people or the environment. Individuals in Class 7 helped by clicking primarily when posts were about animals or the environment, but they very rarely helped in response to posts about people. These results show that the HCTQ makes it possible to identify groups with different patterns of helping by clicking in the areas distinguished in this questionnaire (animals, people, and the environment).

## Discussion

In accordance with the aim of the present studies, we succeeded in developing an innovative instrument assessing the patterns of helping by clicking. Study 1 made it possible to determine the factor structure and psychometric properties of the HCTQ. The structure was confirmed by a confirmatory factor analysis of the data obtained in Study 2. Data collected in Study 2 also made it possible to identify click-to-donate profiles.

The HCTQ is designed to measure three aspects of helping: helping people, helping the environment, and helping animals via social media. This has been the first research study to develop a measure assessing this kind of helping activity, increasingly present in the social media environment (Lucas, 2017). All factors of the new measure have good Cronbach’s alpha reliability coefficients. The results allow us to conclude that the factor structure of the HCTQ fits the theoretical model of helping behavior to an acceptable degree (Aydinli et al., 2013). Additionally, the questionnaire’s items effectively distinguish between different levels of the constructs measured by HCTQ subscales. The response scale also effectively represents the changes in the levels of these constructs. Only the second (*very rarely*) and third (*rarely*) responses may not be sharply differentiated by participants.

From the moral point of view, as far as helping is concerned, a person is obliged to go beyond the interest of their own society or even species (Kopnina et al., 2018). The factors identified in the HCTQ relate to three most frequent areas of aid campaigns: helping people, activities for the natural environment, and helping animals. However, as shown by other studies (Damasio, 2005; Dawkins, 2018; Glomb et al., 2011) and by analyses performed on the data collected in Study 2, people are not always willing to help and not equally willing to help different beneficiaries. The levels of motivation for and engagement in selfless helping may vary across individuals (Rożnowski & Olczykowska, 2016). Using the HCTQ, it is possible to identify groups of people with different patterns of helping by clicking in the areas distinguished in this questionnaire (animals, people, and the environment). This shows that we succeeded in achieving the second goal of the survey, which was to check whether it was possible to identify groups of individuals with different helping patterns using the new tool.

The most radical patterns of helping by clicking are found, on the one hand, in individuals weakly engaged in this form of helping (Class 5), and on the other—in those who always help, regardless of which area their help concerns (Class 8). In the case of the former, it is impossible to clearly determine whether they engage in no helping behaviors at all or whether they do not engage specifically in the kind of helping that involves new media and the clicking options offered there. Despite the high accessibility of social media and the increasing popularity of various kinds of aid campaigns involving these media, justified doubts arise regarding the degree to which this kind of help is effective and the degree to which it actually reaches the needy (Sweatman, 2003). Some people prefer direct face-to-face contact with those they wish to help, and so they do not use social media for this purpose (Jay, 2001).

The individuals who engage in helping by clicking regardless of which area it concerns (Class 8) undoubtedly show high willingness to help. Access to new media enables them to help without putting in much effort, which they frequently do, for example by clicking on a link or sharing contents that include a request (Schattke et al., 2018). According to the opponents of this form of assistance, such passive helping, often without knowing the intention of the person asking for help, cannot be effective because one loses control over where one’s support actually gets (Aydinli et al., 2013; Lucas, 2017). Rather than effective and selfless help, this kind of assistance may sometimes be interpreted as provided for show, in order to raise one’s social prestige and self-esteem or to seek public recognition and admiration (Damasio, 2005; Kopnina et al., 2018). These reflections are part of a larger debate on the issues of helping and altruism and on whether they should bring benefits to the helper, for example in the form of good well-being (Damasio, 2005).

In most of the identified patterns, individuals decided to help when assistance was for the benefit of people (Classes 2 and 3). This seems to be consistent with the sociobiological understanding of altruism, oriented primarily towards helping one’s closest relatives or, in the extended version, towards helping individuals belonging the same species. This ensures that mankind has a greater chance of surviving (Dawkins, 2018). In their study, Ma and Chan (2014) confirmed that, even online, people were more willing to help those from their network of social contacts than complete strangers. On the other hand, helping strangers online on an anonymous basis restricts the possibility of applying the reciprocity principle; as a result, this kind of help seems to be truly selfless (Lucas, 2017) and has the hallmarks of pure altruism (Damasio, 2005).

As mentioned before, the majority of Internet users report engagement in helping other people by clicking. Only in the case of specific few patterns did Internet users help animals (Classes 1 and 6) or animals and the environment (Class 7) more willingly that they helped people. In the case of supporting aid campaigns for the natural environment, the direct beneficiaries of improvement in the environment will also be people. Unfortunately, the social awareness is not particularly high when it comes to engaging in any kind of protective activities for the planet or for other creatures inhabiting it (Goecks et al., 2008). Helping by clicking allows Internet users to provide help even to the most distant corners of the planet or to the animals inhabiting it without leaving their homes. Regrettably, as shown by research, despite a temporary improvement in the situation of animals and the environment, campaigns using new media do not lead to a lasting change of the attitudes and, importantly, behaviors for their benefit (Tiplady et al., 2013).

An unquestionable value of the study reported in this article is the development of the world’s first research measure assessing the patterns of helping by clicking. The measure we have developed has good psychometric properties, enabling its application for research purposes. Additionally, based on the HCTQ, we have developed an eight-class taxonomy of individuals showing various degrees of engagement in helping by clicking, which is an original segmentation of people who engage in helping by means of new media. This information may be useful for organizations helping people, animals, or the environment in choosing appropriate ways of reaching the category of people who might be most willing to support them. It is also part of the increasingly popular movement called effective altruism, which consists in seeking, popularizing, and using the most effective ways of improving the situation in the world (Singer, 2015). It may seem that clicking “Like” is not real help to speak of, but accurately reaching an Internet user willing to provide help of a particular type and guiding him or her through reliable charity institutions can bring tangible results for one of the categories in need of help (people, animals, or the environment; Wallace et al., 2017; Warren et al., 2014).

The development of new media will undoubtedly continue; as a result, the popularity of this form of assistance will be growing, which in turn will increase the need for regular research into this phenomenon by means of useful measures such as the HCTQ. In the future, the universality of this method should be tested in cross-cultural studies. Another interesting direction for research seems to be the search for determinants of belonging to each of the groups distinguished. As suggested by previous studies on altruism, what could be a promising direction for this search is links with variables such as personality traits (Visser & Roelofs, 2011), empathy (Huber & MacDonald, 2012), or mood (Underwood et al., 1977). Research might also test how helping by clicking is influenced by factors such as sex (Brañas-Garza et al., 2018), age (Sparrow et al., 2021), and financial status (Visser & Roelofs, 2011).

The present study has certain limitations. Admittedly, the measure and the taxonomy were developed based on a study with a sample recruited in one country. As mentioned before, research should be expanded to include samples from various cultures; a distinction between predominantly collectivistic and predominantly individualistic cultures might be interesting. In our study, we focused on the positive aspect of helping by clicking. What one must not forget, either, is the pathological forms of altruism, when misguided help can harm both the helper and the environment receiving their assistance (Oakley, 2013). Particularly passive helping, which consists in clicking on a link, very often without checking the real motives behind the aid campaign, may ultimately diverge from the idea of altruistic help (Schattke et al., 2018; Wallace et al., 2017). It should be remembered, too, that new media can be used also to promote active helping (Aydinli et al., 2013; Warren et al., 2014), which is not taken into account in the new measure.

To sum up, the outcome of the present study is a new measure of helping patterns. We have identified three factors in the structure of this measure: helping people by clicking, helping the environment by clicking, and helping animals by clicking. The HCTQ concerns a new and largely unexplored area of helping by means of modern technologies. It reveals people’s motivations for helping. Research into the psychometric properties of the measure supported its reliability, showing that it could be widely used in the study of online behaviors.

## Supplementary Materials

The Supplementary Materials include the following items:

- The research data for Study 1 (Błachnio, 2024S-a)
- The research data for Study 2 (Błachnio, 2024S-b)
- Additional Information: Supplementary materials include the contents of items and statistical analysis scripts executed in SPSS software for Studies 1 and 2. Additionally, they include the results of pairwise comparisons of class membership on each HCTQ subscale level (Błachnio, 2024S-c).

BłachnioA.
PrzepiórkaA.
KotP.
CudoA.
SobolM.
 (2024S-a). The development of the Helping by Clicking Types Questionnaire (HCTQ) – Study 1
[Research data]. PsychOpen. https://hdl.handle.net/20.500.12153/6427
10.5964/ejop.10917PMC1163870039678923

BłachnioA.
PrzepiórkaA.
KotP.
CudoA.
SobolM.
 (2024S-b). The development of the Helping by Clicking Types Questionnaire (HCTQ) – Study 2
[Research data]. PsychOpen. https://hdl.handle.net/20.500.12153/6428
10.5964/ejop.10917PMC1163870039678923

BłachnioA.
PrzepiórkaA.
KotP.
CudoA.
SobolM.
 (2024S-c). Supplementary materials to "The Helping by Clicking Types Questionnaire (HCTQ): The development of a measure to assess different patterns of helping by clicking"
[Materials and Code]. PsychOpen. 10.23668/psycharchives.15179
PMC1163870039678923

## Data Availability

The research datasets to replicate the findings of both studies are publicly available (see Błachnio et al., 2024S-a, Błachnio et al., 2024S-b).
